# Supplementary figures and images for: Designing Genome-Wide Association Studies: Sample Size, Power, Imputation, and the Choice of Genotyping Chip
Source: PLoS Genet. 2009 May 15;5(5):e1000477. doi: 10.1371/journal.pgen.1000477 (PMC2688469; doi:10.1371/journal.pgen.1000477)

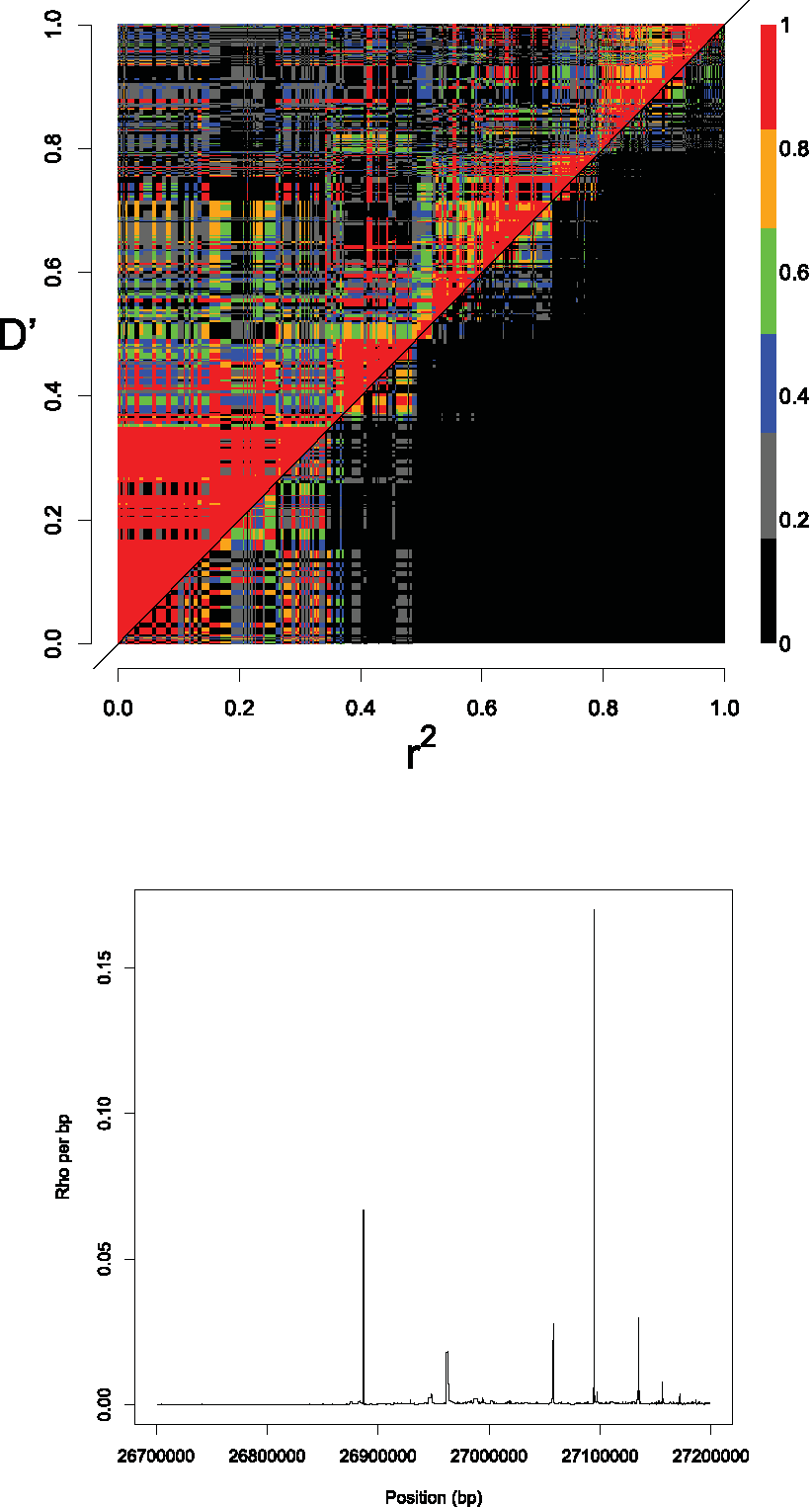

Supplement: Figure S1 — Top plot : Linkage disequilibrium plots across the region : D′ (top left), r2 (bottom right). Bottom plot : Fine scale recombination map ρ across the region. (0.21 MB TIF) [file pgen.1000477.s001.tif]

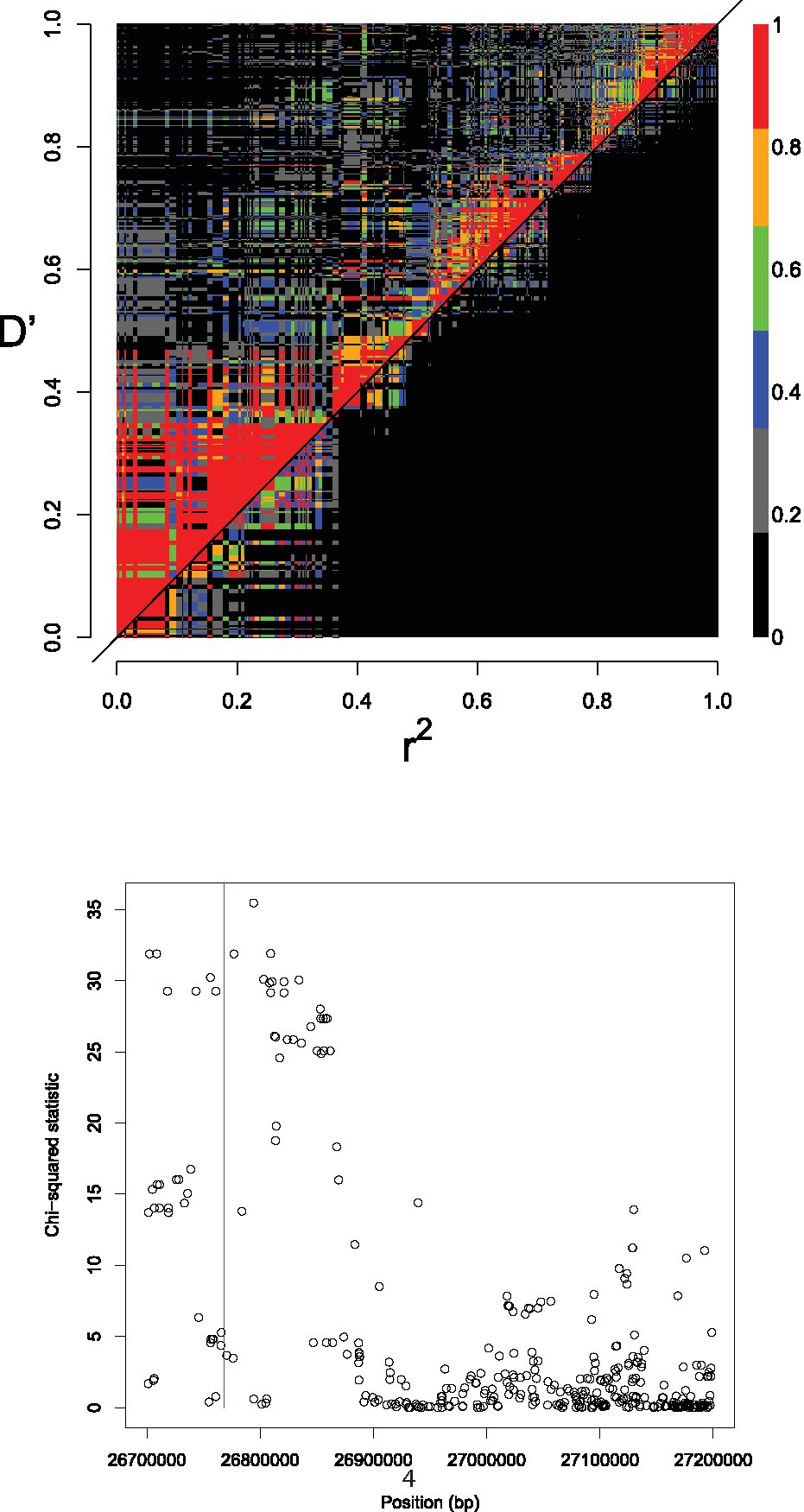

Supplement: Figure S2 — For simulated dataset A (α = 1.3, β = 1.69) the top plot shows D′ and r2 LD measures. The bottom plot shows the χ2 statistic for association across the region. The vertical blue line shows the location of the disease locus. (0.28 MB TIF) [file pgen.1000477.s002.tif]

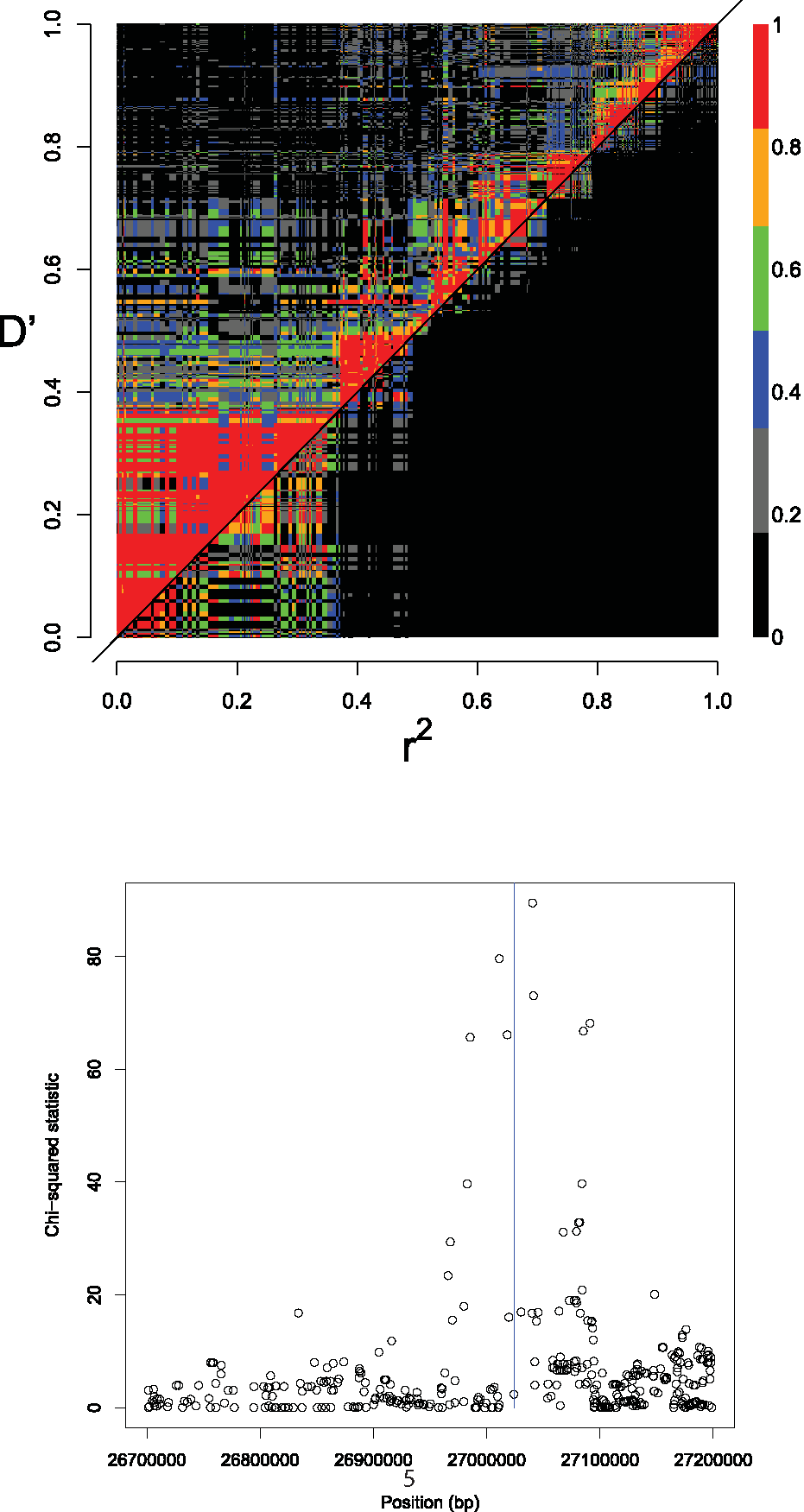

Supplement: Figure S3 — For simulated dataset B (α = 1.5, β = 2.25) the top plot shows D′ and r2 LD measures. The bottom plot shows the χ2 statistic for association across the region. The vertical blue line shows the location of the disease locus. (0.29 MB TIF) [file pgen.1000477.s003.tif]

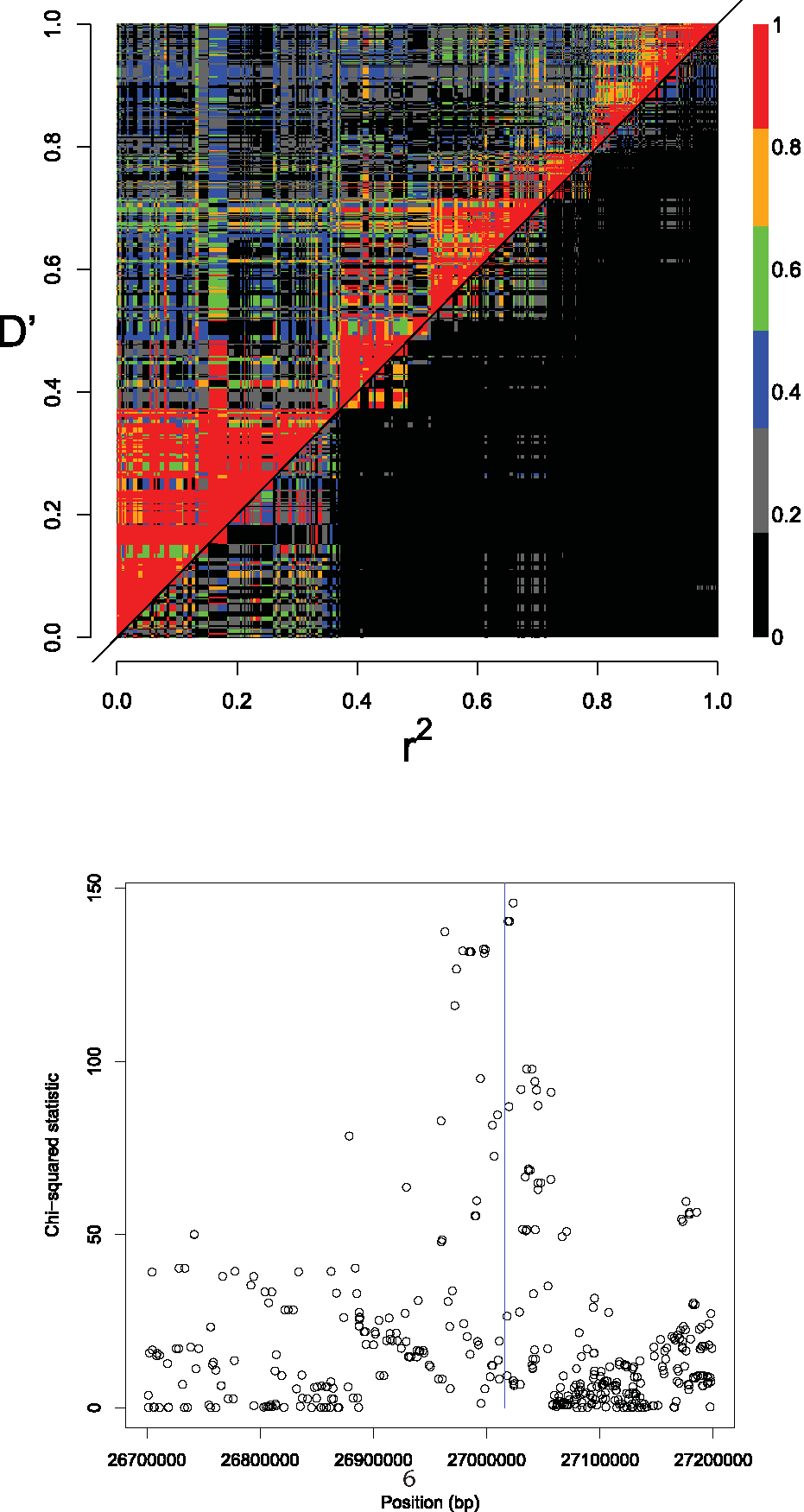

Supplement: Figure S4 — For simulated dataset (α = 1.7, β = 2.89) the top plot shows D′ and r2 LD measures. The bottom plot shows the χ2 statistic for association across the region. The vertical blue line shows the location of the disease locus. (0.30 MB TIF) [file pgen.1000477.s004.tif]
