# Supplementary material for: Designing Genome-Wide Association Studies: Sample Size, Power, Imputation, and the Choice of Genotyping Chip
Source: PLoS Genet. 2009 May 15;5(5):e1000477. doi: 10.1371/journal.pgen.1000477 (PMC2688469; doi:10.1371/journal.pgen.1000477)
Supplement: Text S1 — Supplementary text associated with the main article. (0.06 MB PDF) [file pgen.1000477.s009.pdf]

Supplementary Material

Illustration of HAPGEN

In order to illustrate our approach we used one of the 10 ENCODE regions selected for extensive analysis as part of the International HapMap project [1] in the CEU population. The haplotypes are sampled at 1314 SNPs on chromosome 7 in a 500 kb region between base pair positions 26,699,793 and 27,199,792. The data were collected as genotypes of 30 mother-father-child trios and the program PHASE v2.1 [2] was used to estimate haplotypes and fine-scale recombination rates. Supplementary figure 1 shows two measures of LD (r^2 and D') across the region together with the fine-scale recombination map. This figure clearly shows that there is extensive variation in the correlation structure and underlying recombination rate across the region with spikes or hotspots of recombination demarcating regions of high LD.

We used this dataset to simulate case-control datasets consisting of 1000 case and 1000 control individuals. We simulated 3 new datasets using the disease models (A) $\alpha = 1.3, \beta = 1.69$, (B) $\alpha = 1.5, \beta = 2.25$, (C) $\alpha = 1.7, \beta = 2.89$. On a desktop machine with an AMD Opteron 244 processor and 2Gb of RAM, generating these datasets took 2.4 seconds each.

Supplementary Figures 2-4 show the LD plots and results of a single locus association tests for the three datasets. The locations of the disease loci in each dataset is marked with a blue line. It is clear to see that the LD properties of the simulated datasets closely match those of the original data. Also, as expected the signal of association increases from models A-C and that the regions in which there is evidence of association correlated well with regions of high LD in the original dataset.

Comparing HAPGEN to coalescent simulations

The simulations in the section above illustrate graphically that HAPGEN is able to produce case-control datasets with realistic levels of LD. In order to assess in a more quantitative fashion how well HAPGEN does at creating appropriate levels of LD we compared it to the widely used coalescent simulator called MS (available from `http`). It should be noted that coalescent simulators are not able to simulate genotype data conditional upon a known set of haplotype data in the way that HAPGEN does so they do not provide an alternative solution. The simulations involved the following steps.

1. We used MS to simulate 620 haplotypes over a 1Mb region. The recombination rate was set to 1CM/Mb. The mutation rate in MS was set to 600. We used the first 4 simulated chromsomes to ascertain SNPs and this resulted in approximately 1000 SNPs per simulation in the region.

2. We then used the first 120 haplotypes to act as a panel of genetic variation. We determied the set of SNPs (denoted T) that tagged all common SNPs (MAF $\geq 5\%$) in the panel and the set of SNPs that are common minus the tag set (denoted S).

3. We then measured the fraction of SNPs in the set S that are tagged by the set T in the remaining set of 500 haplotypes simulated by MS.

4. We then simulated a new set of 500 haplotypes using the HAPGEN and the panel of 120 haplotypes and calculated the fraction of common SNPs captured by the tag set.

We repeated steps 1-4 300 times and calculated the average fraction of tagged common variation for both MS and HAPGEN as 0.867 and 0.854 respectively. The close agreement of these fractions shows that HAPGEN is simulating a realistic amount of genetic variation.

Comparison of genotyping chips

In order to compare the power of the different genotyping chips taking into account their various prices we obtained quotes from the following service providers

Almac `http://www.almacgroup.com`
Medical Solutions `http://www.medical-solutions.co.uk`
Affymetrix `http://www.affymetrix.com`
The Broad Institute `http://www.broad.mit.edu`
Dnavision `http://www.dnavision.be`
Atlas Biolabs `http://www.atlas-biolabs.de`
Decode `http://www.decode.com`

References

[1] The International HapMap Consortium (2005) A Haplotype Map of the Human Genome. Nature 437: 1299-320.

[2] Stephens M, Donnelly P (2003) A comparison of bayesian methods for haplotype reconstruction from population genotype data. Am J Hum Genet 73: 1162–1169.
